# Antithrombotic Effect of *Chenopodium album* L. Extract and Its Fractions via Regulating TLRs and the Downstream MAPKs and PI3K/AKT Signaling Pathways in Zebrafish

**DOI:** 10.3390/ijms26052118

**Published:** 2025-02-27

**Authors:** Xiyue Wang, Miaoyunhuan Wang, Yuqing Dong, Shuqing Yu, Shanshan Zhang, Pinghua Sun, Lu Wang, Jibin Liu, Houwen Lin, Xinhui Pan, Xiaobin Li

**Affiliations:** 1 Laboratory of Xinjiang Phytomedicine Resource and Utilization, Ministry of Education, School of Pharmaceutical Sciences, Shihezi University, Shihezi 832002, China; wangxiyue19990430@126.com (X.W.); pinghuasunny@163.com (P.S.); 13677546568@163.com (L.W.); 2 Engineering Research Center of Zebrafish Models for Human Diseases and Drug Screening of Shandong Province, Biology Institute, Qilu University of Technology (Shandong Academy of Sciences), 28789 East Jingshi Road, Jinan 250103, China; wangmiaoyunhuan@163.com (M.W.); dongyuqing777@163.com (Y.D.); 18954178134@163.com (S.Y.); zhangss@sdas.org (S.Z.); liujibin310@163.com (J.L.); franklin67@126.com (H.L.); 3 State Key Laboratory of Bioactive Molecules and Druggability Assessment, College of Pharmacy, Jinan University, Guangzhou 510632, China; 4 Research Center for Marine Drugs, State Key Laboratory of Oncogenes and Related Genes, Department of Pharmacy, School of Medicine, Shanghai Jiao Tong University, Shanghai 200127, China

**Keywords:** antithrombotic effect, *Chenopodium album* L., zebrafish, network pharmacology, transcriptomics, RT-qPCR

## Abstract

*Chenopodium album* L., as a folkloric herb, is traditionally used to treat poisonous insect bites, vitiligo, and other ailments. However, its impact on thrombosis remains unknown. In this study, we discovered that the ethanol extract of *C. album* exhibited a remarkable antithrombotic effect using a zebrafish thrombosis model for the first time. Activity evaluation showed that fraction CA-C could improve thrombus aggregation in the caudal vein, increase blood return in the heart, and alleviate the slowing of blood flow compared with those in the model group. Then, analysis by ultra-high performance liquid chromatography-quadrupole time-of-flight tandem mass (UPLC-Q-TOF-MS) identified 58 constituents of CA-C, with most of them belonging to flavonoids, alkaloids, and steroidal saponin components. Moreover, using a comprehensive strategy of network pharmacological analysis, transcriptomic assay, and RT-qPCR validation, we found that CA-C could mediate the TLR’s signaling pathway and its downstream MAPKs and PI3K/AKT signaling pathways to exert an antithrombotic effect. This study broadens the clinical application of plant *C. album* and provides new insight into the chemical profile, pharmacodynamics, and potential mechanisms of CA-C as candidate agents for treating thrombosis.

## 1. Introduction

As a common trigger of cardiovascular disease, thrombosis can result in partial or complete blockages of blood vessels, causing ischemic heart disease, stroke, and atherosclerosis. Thrombotic diseases have become the leading cause of death due to diseases globally [1,2]. Factors contributing to thrombus formation include platelet adhesion, activation, secretion, and aggregation, as well as thrombin generation and fibrin formation triggered by tissue factors, which lead to slowed blood flow, vascular occlusion, and insufficient blood supply to the heart. Platelets, during activation, undergo adhesion and aggregation reactions to a certain extent, which can promote thrombosis and, in turn, lead to myocardial ischemia, ischemic stroke, and other cardiovascular disease. Besides, thrombus formation can aggravate the degree of atherosclerosis, and atherosclerosis can also induce thrombus formation. They interact with each other and jointly participate in the occurrence and development of many cardiovascular diseases [3,4]. Platelets are related to many physiological functions, primarily mediating hemostasis and thrombosis, and play an essential role in immunoinflammatory processes. Platelets express multiple receptors that result in platelet functional responses [5]. Toll-like receptors (TLRs) are natural immune receptors that participate in cellular immune responses and inflammatory responses by recognizing ligands, and there is a strong link between inflammatory responses and thrombosis [6,7]. Research consistently indicates that platelet TLRs play a crucial role in platelet activation and aggregation, and myeloid differentiation primary response protein 88 (MyD88) expressed by platelets mediates downstream signaling of most TLRs. Furthermore, the mitogen-activated protein kinases (MAPKs) signaling pathway, a relevant pathway of platelet activation, regulates multiple cell biological responses. An absence of external stimulation results in a quiescent MAPK signaling pathway. Upon stimulation, it undergoes activation via a cascade of phosphorylation events, leading to platelet activation and aggregation, thereby initiating an inflammatory response. Furthermore, it has the capacity to amplify the inflammatory effect through additional cascade reactions, resulting in enhanced platelet activation [8]. Human platelets express four isoforms of phosphoinositide-3-kinase (PI3K), with PI3Kβ being crucial for platelet activation signaling, while the other isoforms support this function. AKT, a key downstream protein kinase in the PI3K/AKT pathway, has three isoforms (AKT1, AKT2, and AKT3) in platelets. PI3K enzyme inhibitors help prevent thrombosis and atherosclerosis in the cardiovascular system [9].

Network pharmacology is a new discipline of multi-target drug molecular design based on systems biology theory. It emphasizes the multi-pathway modulation of signaling pathways to improve drug therapeutic efficacy. Significantly, network pharmacology screens and constructs networks at different levels to explain the mechanisms by which drugs exert beneficial effects on diseases [10]. Traditional Chinese medicines (TCMs) usually contain multiple chemical components that can act on multiple biological targets. Network pharmacology has been widely used to identify effective targets and components of TCMs [11]. Obtaining biological data through “omics” technology has become very popular in recent years. Among them, transcriptomics provides advanced technology for systems such as in biology research. Importantly, transcriptomics is the study of gene expression from the RNA level, which helps to identify many differentially expressed genes and the signaling pathways they regulate [12].

*Chenopodium album* L. is a wild vegetable resource with rich nutritional value and extensive medicinal value. Some studies have shown that young stems and leaves and mature plants of *C. album* contain V_C_ and *β*-carotene, and both leaves and seeds contain V_A_ and V_C_ [13]. The protein content of *C. album* is very rich and is among the highest in leafy and stem vegetables [14]. It also contains a variety of essential and non-essential amino acids [15]. Additionally, *C. album* can treat a wide range of diseases and has high medicinal research and development value. For example, researchers have discovered that the methanolic extract of *C. album* leaves had a growth inhibitory effect against Ehrlich ascites carcinoma cells, showing in vivo anticancer activity [16]. The acetone extract of the above-ground parts of *C. album* showed an anti-arthritic activity, and the butanol extract could increase mucus content in the stomach and alleviate the symptoms of gastritis [17]. In vitro, ethanolic extracts of *C. album* can inhibit the growth of *Pseudomonas aeruginosa*, *Candida albicans*, and *Helicobacter pylori* [18]. Furthermore, *C. album* also has hepatoprotective [19] and anthelmintic [20] activities.

Arachidonic acid (AA), catalyzed by cyclooxygenase, generates thromboxane A2, which induces the release of adenosine diphosphate from platelets and triggers platelet aggregation, and its thrombus-inducing mechanism is related to the arachidonic acid metabolic pathway [21]. Zebrafish, with its transparent embryos, high fecundity, rapid embryonic development, and high homology to mammalian species, has become a vital link bridging cellular and mammalian models in international drug screening and evaluation systems [22]. Extrinsic and intrinsic coagulation factors and various platelet surface receptors in zebrafish blood or platelets make zebrafish ideally suited for hematological studies and drug screening of relevant activities [23]. Zebrafish has also been widely used in the efficacy evaluation and mechanisms research of antithrombotic agents [24]. In this study, we assessed the antithrombotic effect of CA and its fractions and further explored the potential targets and mechanisms of CA-C using the AA-induced thrombus model in zebrafish.

In the present study, through extensive activity screening using zebrafish models, we first found that *C. album* extract (CA) can exert significant antithrombotic activity. Later, CA-C was determined as the main active fraction by arachidonic acid (AA)-induced zebrafish thrombus model. The chemical analysis of CA-C was performed using ultra-high performance liquid chromatography-quadrupole time-of-flight tandem mass (UPLC-Q-TOF-MS). Subsequently, a comprehensive investigation into the potential mechanisms underlying the antithrombotic activity of CA-C was conducted through an integrated approach combining network pharmacology and transcriptomics. These findings were further validated using RT-qPCR analysis.

## 2. Results

### 2.1. Antithrombotic Effect of CA on Zebrafish

**Figure 1:**
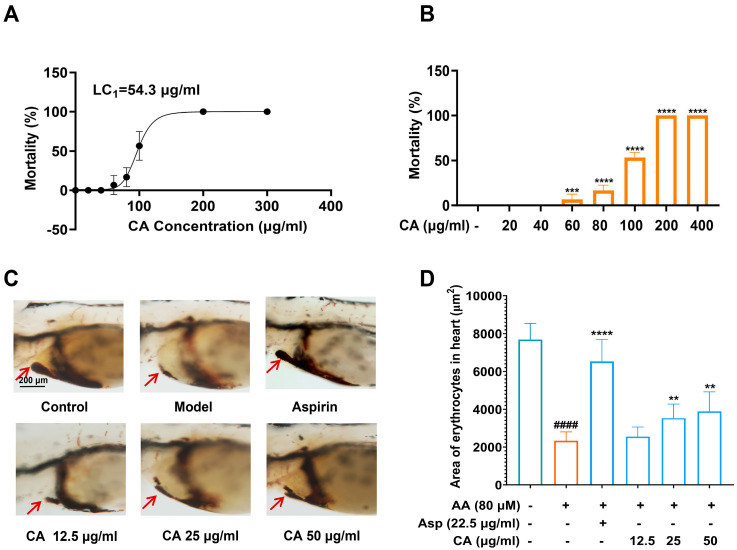
Investigation of the safe concentration of CA for zebrafish larvae and evaluation of its antithrombotic activity. (**A**,**B**) Mortality curves of zebrafish after CA treatment at different concentrations and mortality rate at 24 hpe (**** *p* < 0.0001 and *** *p* < 0.001 compared to the control group); (**C**) typical pictures of erythrocyte staining phenotypes in the heart of zebrafish in each group, with red arrows pointing to the zebrafish heart site in the figure; (**D**) histogram of the stained area of erythrocytes in the zebrafish hearts of each group. ^####^ *p* < 0.0001 vs. the control group, ** *p* < 0.01 and **** *p* < 0.0001 vs. the model group.

Before the activity evaluation, the safe dose of CA for zebrafish larvae was investigated first. The results are shown in Figure 1A,B. CA at the concentration of 40 μg/mL and below had no significant toxicity to the development of zebrafish larvae. Zebrafish mortality rates at 96 hpf were used to plot the CA lethal curve, and the minimum lethal concentration of CA (LC_1_) was calculated to be 54.3 μg/mL. Based on the above results, three concentrations, 12.5, 25, and 50 μg/mL, which were lower than LC_1_, were selected for the subsequent experiments to evaluate the antithrombotic activity of CA.

We used the AA-induced zebrafish thrombus model to test the antithrombotic effect of CA. As shown in Figure 1C,D, thrombus formation of zebrafish in the model group leads to a decrease in venous return of zebrafish, which is manifested by a reduction of erythrocytes in the zebrafish heart region compared with that in the control group. CA was able to significantly reverse AA-induced erythrocyte reduction in the zebrafish heart at concentrations of 25 and 50 μg/mL, suggesting that CA has a remarkedly antithrombotic activity.

### 2.2. Antithrombotic Effect of Fractions from CA on Zebrafish

**Figure 2:**
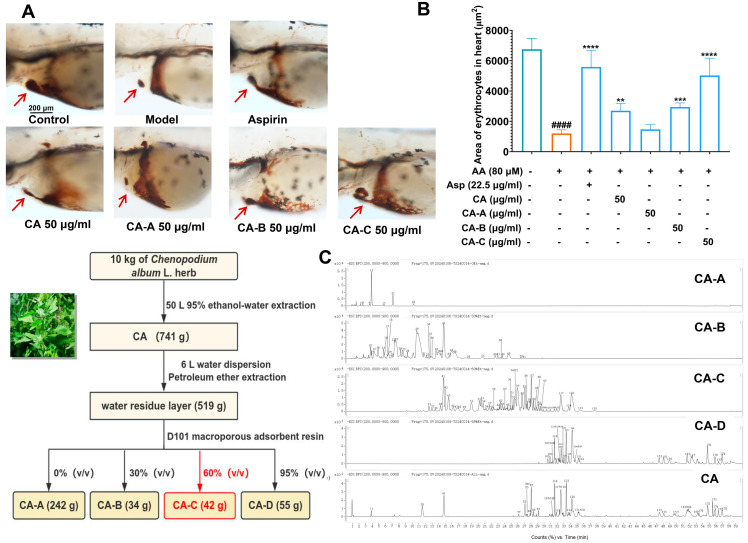
Evaluation of antithrombotic activity and compositional analysis of CA, CA-A~CA-D; (**A**) typical pictures of erythrocyte staining phenotypes in the heart of zebrafish of each group, with red arrows pointing to the zebrafish heart; (**B**) histogram of the stained area of erythrocytes in the zebrafish heart of each group. ^####^ *p* < 0.0001 vs. the control group, ** *p* < 0.01, *** *p* < 0.001, **** *p* < 0.0001 vs. the model group. (**C**) The base peak chromatograms of CA, CA-A~CA-D using UPLC-Q-TOF-MS in negative-ion modes.

For the purpose of identifying the active components of CA, we obtained four fractions, CA-A to CA-D, from CA by macroporous resin chromatography and screened their antithrombotic activity. As shown in Figure 2A–C, compared with the model group, CA, CA-B, and CA-C groups were able to inhibit AA-induced erythrocyte reduction in the heart region of zebrafish, which showed strong antithrombotic activity. Among them, the activity of CA-C was much more significant than that of others. In addition, the zebrafish in the CA-D group were all dead, which exhibited an obvious toxicity of CA-D to zebrafish.

### 2.3. UPLC-Q-TOF-MS Analysis of Fractions from CA

**Figure 3:**
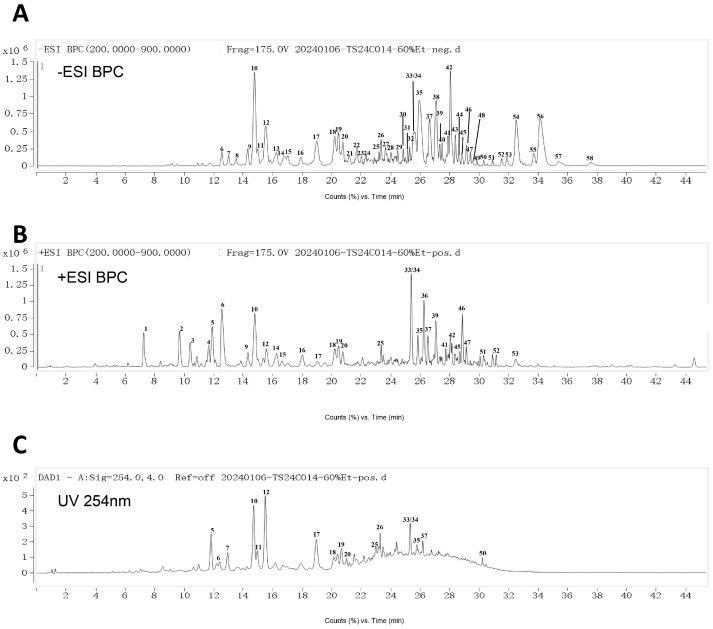
Analyses of chemical constituents of CA-C using UPLC-Q-TOF-MS. (**A**–**C**) Base peak chromatograms of CA-C in the negative and positive-ion MS modes, and LC diagram at 254 nm, respectively.

Chemical components of CA and CA-A to CA-D were characterized by UPLC-Q-TOF-MS (Figure 2C). Combining information from multistage mass spectrometry and high-resolution mass spectrometry databases of natural products, 178 compounds were detected in the samples, and most of them were identified as flavonoids, alkaloids, steroids, or saponins. In CA-C, the peak area of alkaloids accounted for 28.21% of the total peak area, steroidal saponins accounted for 25.10%, flavonoids accounted for 12.23%, fatty acids accounted for 10.12%, and other types of components accounted for 24.34%. A total of 58 compounds were detected from CA-C (Figure 3A–C and Table 1), including 33 compounds which were identified as 9 saponins (Serial Nos. 9, 10, 15, 18, 19, 25, 33, 46, and 49), 7 alkaloids (Serial Nos. 5, 14, 22, 24, 34, 37, and 38), 6 flavonoids (Serial Nos. 8, 11, 12, 17, 20, and 21), 6 fatty acids (Serial Nos. 39–44), 1 triterpene (Serial No. 56), 1 sugar derivative (Serial No. 6), 1 amino acid derivative (Serial No. 3), and 2 peptides (Serial Nos. 52 and 53), and 25 alkaloids or saponins whose structures were not able to be identified temporarily. The mass spectrometry data of these unidentified structures were not found to be matched with data in databases and the literature, suggesting that they are most likely new compounds. Of course, a systematic chemical investigation of CA-C is needed to further confirm this conjecture. Besides, forskoditerpenoside C (Serial No. 56), spinasaponin A (Serial No. 46), ajugasalicioside F (Serial No. 25), and multiple ochangoside analogues (Serial Nos. 14, 24, 34, 37, and 38) were detected for the first time from the plant of *C. album*. In light of the above results, alkaloids, steroidal saponins, and flavonoid glycosides are the major constituents of CA-C, and further systematic chemical isolation is needed to investigate the unidentified chemical composition.

### 2.4. Antithrombotic Activity of CA-C

**Figure 4:**
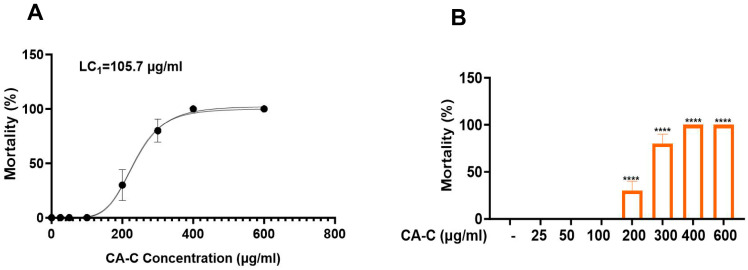
Investigation of the safe concentration of CA-C for zebrafish larvae mortality curves (**A**) and mortality rate (**B**) of zebrafish after CA-C treatment at different concentrations (**** *p* < 0.0001 compared to the control group).

Before the activity evaluation, the safe dose of CA-C for zebrafish larvae was investigated first. The results are shown in Figure 4A,B. CA-C at the concentration of 100 μg/mL and below had no significant toxicity to the development of zebrafish larvae. Zebrafish mortality rates at 96 hpf were used to plot the CA-C lethal curve, and the minimum lethal concentration of CA-C (LC_1_) was calculated to be 105.7 μg/mL.

**Figure 5:**
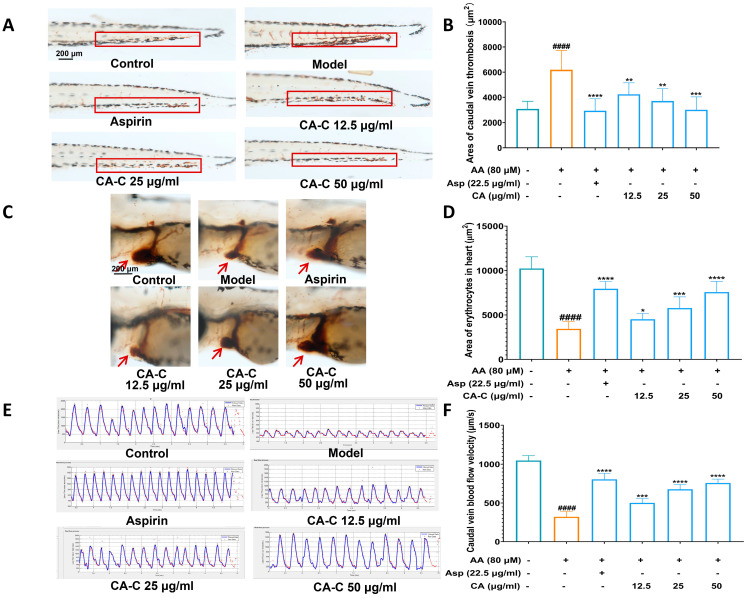
Evaluation results of antithrombotic activity for CA-C at gradient concentrations. (**A**) A typical picture of zebrafish caudal thrombus staining, with the area of zebrafish caudal thrombus counted in the red line box; (**B**) histogram of thrombus area in the caudal vein of zebrafish; (**C**) typical pictures of erythrocyte staining phenotypes in the heart of zebrafish, with red arrows pointing to the zebrafish heart site; (**D**) histogram of the stained area of erythrocytes in the zebrafish heart; (**E**) blood flow dynamics of the caudal vein in zebrafish; (**F**) plot of blood flow velocity in caudal vein of zebrafish. ^####^ *p* < 0.0001 vs. the control group, * *p* < 0.05, ** *p* < 0.01, *** *p* < 0.001, **** *p* < 0.0001 vs. the model group.

We evaluated the antithrombotic activity of CA-C with the gradient concentration in various aspects. As shown in Figure 5A,B, AA caused the formation of a thrombus in the caudal vein of zebrafish, while CA-C can inhibit AA-induced thrombus occlusion with a concentration-dependent manner in the concentration ranges of 12.5–50 μg/mL. Since the formation of the caudal thrombus in zebrafish leads to a decrease in its blood return to the heart, CA-C at above different concentrations could significantly reverse AA-induced erythrocyte reduction in the zebrafish heart (Figure 5C,D). Besides, CA-C also significantly alleviated the AA-induced slowing of blood flow in zebrafish, as shown in Figure 5E,F. Taken together, these results confirm that CA-C has a significant antithrombotic effect.

### 2.5. Results of Network Pharmacology Analysis

**Figure 6:**
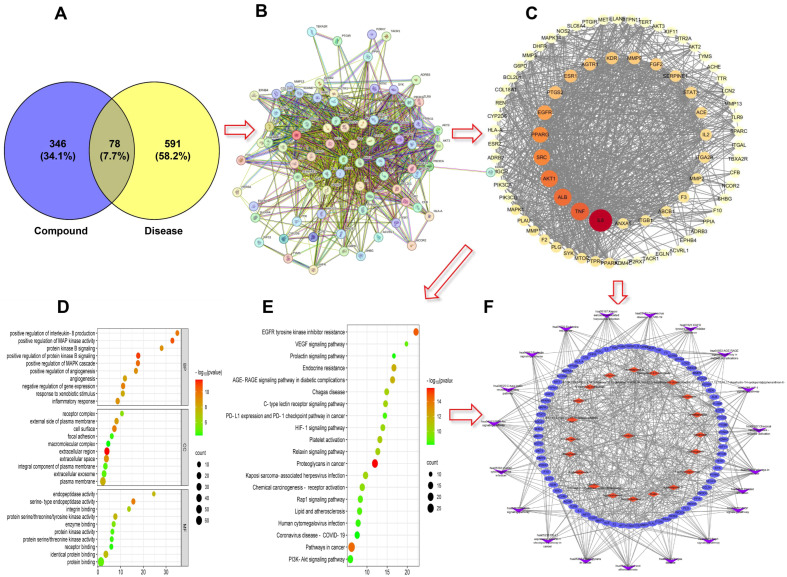
Results of network pharmacology analysis. (**A**) A Venn diagram showing the intersection of CA-C component-related targets and thrombosis-related targets; (**B**,**C**) a PPI network of CA-C in the treatment of thrombosis; (**D**) GO enrichment result; (**E**) KEGG pathway enrichment result; (**F**) “component–target–pathway” visualization network diagram.

We employed a network pharmacology approach to project the material basis and potential mechanisms underlying the antithrombotic activity of CA-C. Our first step was to collect data from various databases, and, thereby, we identified 424 targets related to drug ingredients and 669 targets related to thrombotic diseases. Based on Venn analysis (Figure 6A), 78 potential targets of CA-C associated with thrombosis were screened. The potential targets were generated into a PPI network graph with 72 nodes and 902 edges using the STRING online database platform (Figure 6B). Subsequently, we utilized Cytoscape 3.9.1 software to analyze the topological parameters of the protein-protein interaction (PPI) network via the analyze network function. Targets were prioritized based on their degree values, with higher values indicating greater significance in the biological process. The top ten targets identified were IL-6, TNF, ALB, AKT1, SRS, PPARG, EGFR, PTGS2, ESR1, and AGTR1 (Figure 5 and Figure 6).

By GO enrichment analysis (Figure 5 and Figure 6), 231 GO items were screened based on *p* ≤ 0.01 and FDR ≤ 0.05, of which 164 items were related to biological process (BP), 38 items were related to cell component (CC), and the other 29 items were related to molecular function (MF). The main BPs involved were positive regulation of protein kinase B signaling, positive regulation of MAP kinase activity, positive regulation of MAPK cascade, positive regulation of angiogenesis, inflammatory response, and other processes. The enriched CC mainly included receptor complex, extracellular, cell surface, etc., and relevant molecular function (MF) mostly involved serine-type endopeptidase activity, integrin binding, receptor binding, etc. Many of the GO enrichment terms above are related to “kinase binding” and “inflammatory response”. By the KEGG enrichment analysis, 76 signaling pathways were screened based on *p* ≤ 0.01 and FDR ≤ 0.05, of which the top 20 were shown in Figure 6E. The enriched signaling pathways involve platelet activation, PI3K-Akt signaling pathway, C-type lectin receptor signaling pathway, VEGF signaling pathway, etc., and many of the pathways are closely related to platelet activation and regulation.

The “Component-target-pathway” network diagram is shown in Figure 6F. The results showed that the core chemical constituents of CA-C exerting antithrombotic activity were (2S,3R,10R,13R,14S)-2,3,14-trihydroxy-10,13-dimethyl-17-[(2R,3R,5R)-2,3,5-trihydroxy-5,6-dimethylheptan-2-yl]-2,3,4,5,9,11,12,15,16,17-decahydro-1H-cyclopenta[a]phenanthren-6-one, 9,12,13-trihydroxy-10,15-octadecadienoic acid, N-acetyl-DL-tryptophan, forskoditerpenoside C, kaempferol-3-O-rutinoside, azukisaponin III, *β*-ecdysone, turkesterone, rutin, and xanthorhamnin, etc., which interacted with multiple thrombo-associated targets. These also suggested that components in CA-C may have synergistic effects on these targets to exert pharmacological effects.

### 2.6. Transcriptomics Analysis

**Figure 7:**
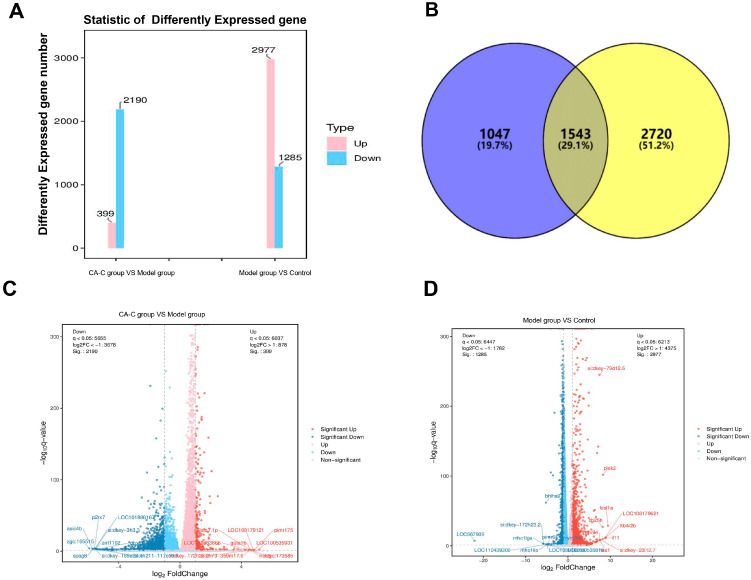
Effect of CA-C on transcriptome analysis in thrombotic zebrafish. (**A**) Histogram of differentially expressed gene (DEG) statistics; (**B**) DEG Venn diagram, different colors represent different combinations of comparisons; (**C**) volcano plot of DEGs between the model and control groups (*p* < 0.05 & |log2FC| > 1); (**D**) volcano plot of DEGs between the CA-C and model groups (*p* < 0.05 & |log2FC| > 1).

mRNA profiles of the control group, model group, and CA-C administration group were determined using transcriptome sequencing. Figure 7A showed a significant difference in genes between the three groups, suggesting that the modeling of AA-induced zebrafish thrombus was successful and the analytical method had good stability and reproducibility. DEGs were identified employing pairwise comparisons (*p* < 0.05, ǀlog2FCǀ > 1) of the control, model, and CA-C administration groups, and the statistics of the number of DEGs among the different comparison groups are shown in Figure 7B. There were 1543 co-DEGs in total between the comparisons of the model group vs. the control group and the CA-C group vs. the model group. As can be seen from the volcano plots in Figure 7C,D, 2977 genes were up-regulated, and 1285 genes were down-regulated in the comparison of the model group vs. the control group, while 399 genes were up-regulated and 2190 genes were down-regulated in the comparison of the CA-C group vs. the model group. Among them, the expression of 1543 DEGs showed opposite trends in the two comparisons above.

**Figure 8:**
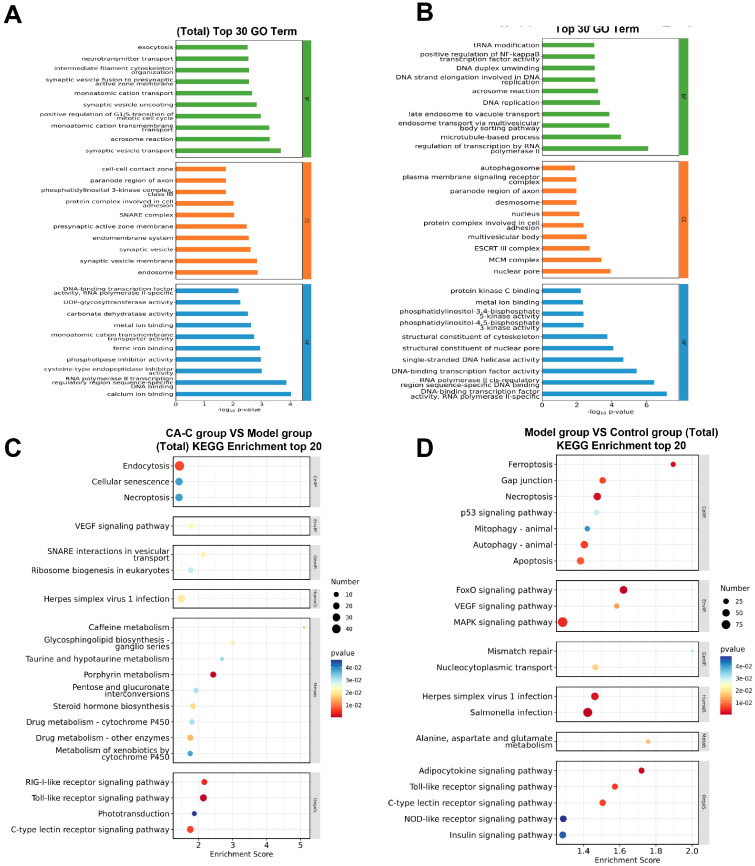
Results of GO and KEGG enrichment analysis in transcriptome assay. (**A**) BarPlot of GO enrichment analysis for DEGs between the model and CA-C groups; (**B**) BarPlot of GO enrichment analysis for DEGs between the model and control groups; (**C**) ScatterPlot of KEGG enrichment analysis for DEGs between the model and CA-C groups; (**D**) ScatterPlot of KEGG enrichment analysis for DEGs between the model and control groups.

**Figure 9:**
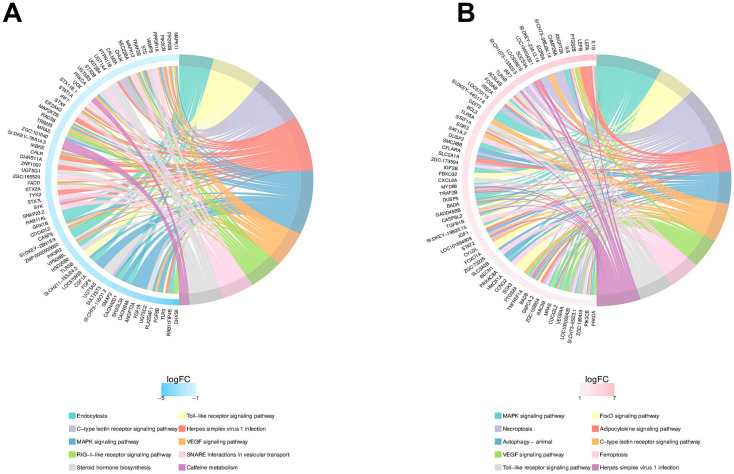
Chord diagram of KEGG enrichment analysis of differential genes. (**A**) KEGG Chord diagram for the DEGs down-regulated between the CA-C and model groups; (**B**) KEGG Chord diagram for the DEGs up-regulated between the model and control groups.

Next, GO functional enrichment analysis and KEGG pathway enrichment analysis were performed with 1543 DEGs. By the GO enrichment analysis, the top 10 GO entries respectively related with BP, CC, and MF are shown in Figure 8A,B. Compared to the model group, altered BP-related items in the CA-C-treated group included exocytosis, neurotransmitter transport, intermediate filament cytoskeleton organization, etc. For CC-related items, CA-C mainly acted on protein complex involved in cell adhesion and phosphatidylinositol 3-kinase (PI3K) complex class IB, paranode region of the axon, etc., while for MF-related items, CA-C mainly regulated phospholipase inhibitor activity, cysteine-type endopeptidase inhibitor activity, etc. The KEGG pathway enrichment analysis (Figure 8C,D) showed that CA-C could exert antithrombotic effects by affecting the TLRs signaling pathway, C-type lectin receptor signaling pathway, RIG-I-like receptor signaling pathway, etc. According to the Chord diagram of KEGG enrichment analysis (Figure 9A,B), the genes associated with the MAPK signaling pathway, the toll-like receptor signaling pathway, the VEGF signaling pathway, and so on, were significantly up-regulated in response to AA induction, which was later reversed by CA-C treatment. These genes included *tlr5*, *tlr3*, *pik3cb*, *mapk11*, *myd88*, *map2k2b*, *mapk13*, *tyk2*, *stat1a*, *irf7*, *chuk*, and so on.

### 2.7. RT-qPCR Analysis

**Figure 10:**
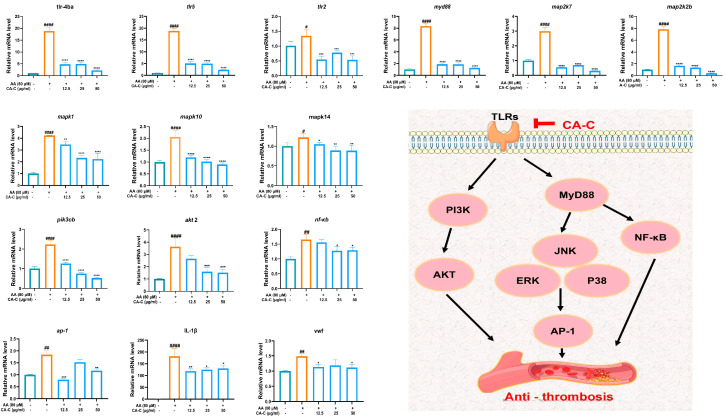
Effect of CA-C on the expression levels of thrombosis-related genes in zebrafish. ^#^ *p* < 0.05, ^##^ *p* < 0.01, **^####^** *p* < 0.0001 vs. the control group, * *p* < 0.05, ** *p* < 0.01, *** *p* < 0.001, **** *p* < 0.001 vs. the model group.

Combining the results of network pharmacology and transcriptomics analysis, we selected genes in the TLR signaling pathway and their downstream MAPK and PI3K/AKT signaling pathways for RT-qPCR validation. The experimental results, as illustrated in Figure 10, demonstrated that AA significantly up-regulated the expression of the genes *tlr2*, *tlr4ab*, *tlr5*, and *myd88* in zebrafish compared to the control group. However, the expression levels of these genes were markedly reduced following treatment with CA-C at concentrations of 12.5, 25, and 50 μg/mL. These findings substantiated the inhibitory effect of CA-C on the TLR signaling pathway. For the MAPKs and PI3K/AKT signaling pathways, we found that the transcript levels of genes *mapk1, mapk14, mapk10, map2k2b, map2k7, akt2*, and *pik3cb* increased in zebrafish after AA induction, whereas CA-C intervention significantly down-regulated their expression levels. Additionally, we also verified the expression levels of nuclear transcription factors *nf-κb* and *ap-1*, *IL-1β,* and *vwf*, and the results showed that CA-C could down-regulate the expression levels of them compared with those in the model group.

## 3. Discussion

During the long-term research, *C. album* has been proved to possess a variety of biological activities. At present, we report the first demonstration of a significant therapeutic effect of *C. album* on thrombosis in the AA-induced zebrafish model. In order to further elucidate the active substance and underlying mechanism of antithrombotic activity for *C. album*, we continue to carry out six aspects of works: (1) obtaining the active fractions responsible for antithrombotic effect in the ethanolic extract of *C. album* (CA) by chromatography of macroporous adsorbent resin together with zebrafish model; (2) utilizing UPLC-Q-TOF-MS to reveal the chemical profile of CA and fractions CA-A–D; (3) systematical evaluation of the antithrombotic effect for active fraction CA-C by zebrafish with several indexes; (4) network pharmacology analysis to infer the possible action mechanism of CA-C and its antithrombotic material basis; (5) assessing the transcriptional level changes of zebrafish thrombus model after CA-C administration through transcriptomics analysis; (6) experimental verification of the potential mechanisms for CA-C employing RT-qPCR assay.

UPLC-Q-TOF-MS analysis detected 58 compounds in CA-C, and 33 structures were tentatively identified among them. The remaining 25 compounds were determined as alkaloids or saponins, but their structures can’t be identified by the mass spectrometry data. So, there may be some novel compounds in CA-C that are worth isolating and purifying next. The primary compounds in fraction CA-C included alkaloids, sterols or saponins, and flavonoid glycosides. in particular, this is the first discovery of compounds forskoditerpenoside C, spinasaponin A, ajugasalicioside F, and various ochangoside analogues from herb *C. album*. Multiple studies have revealed the antithrombotic effects of structure types above and their mechanisms. Alkaloids were found to have good antiplatelet aggregation activity. Berberine significantly inhibited the PI3K/AKT pathway and also inhibited ADP-induced activation of integrin α_IIb_β_3_ and platelet reactions, which in turn inhibited thrombosis [25]. Many plants are rich in steroidal or saponin components that also have essential therapeutic value for thrombotic disorders. *Allium Mavrostomos* saponins have been shown to inhibit the CD40L-induced platelet activation response, which depends on the activation of PI3K/AKT and MAPK pathways [26]. Besides, many natural products of flavonoids were proven to have antithrombotic activity. For example, hesperidin exhibited antithrombotic activity by inhibiting the related gene expressions of TXA2 synthase and TXB2 synthase [27]. Internationally, the zebrafish models have become an important link bridging cellular and mammalian models due to their transparent embryos, rapid embryonic development, and high homology with mammals [22]. The presence of extrinsic and intrinsic coagulation factors and a variety of platelet surface receptors in zebrafish blood or platelets makes zebrafish ideally suited for hematological studies and drug screening [23]. Zebrafish has also been widely used in the evaluation of the efficacy and mechanisms of research of antithrombotic agents [24,28]. Through the use of the thrombus model in zebrafish, we assessed the antithrombotic effect of CA and its fractions and further explored the potential targets and mechanisms of CA-C.

The research on mechanisms of CA-C also integrated strategies of network pharmacology, transcriptomics, and RT-qPCR analysis. GO enrichment analysis of network pharmacology revealed that many target genes of ingredients in CA-C associated with thrombosis are closely related to “positive regulation of kinase activity”, such as positive regulation of MAP kinase activity, positive regulation of MAPK cascade, etc. The KEGG pathway involved the C-type lectin receptor signaling pathway, PI3K/Akt signaling pathway, VEGF signaling pathway, platelet activation, and so on, which are closely related to platelet regulation and antithrombotic formation. In the “component-target-pathway” network (Figure 6F), steroids or saponins and flavonoids interacted with multiple thrombosis-related targets (such as (2S,3R,10R,13R,14S)-2,3,14-trihydroxy-10,13-dimethyl-17-[(2R,3R,5R)-2,3,5-trihydroxy-5,6-dimethylheptan-2-yl]-2,3,4,5,9,11,12,15,16,17-decahydro-1H-cyclopenta[a]phenanthren-6-one, kaempferol-3-O-rutinoside, azukisaponin III, forskoditerpenoside C, β-ecdysone, turkesterone, rutin and xanthorhamnin, etc.), and these compounds also possessed a high content by the UPLC-Q-TOF-MS compositional analysis of CA-C (Table 1). The results presented above substantiated the multi-components, multi-targets, and synergistic properties of CA-C. Transcriptomics is an essential methodology for examining gene expression in vivo, enabling the identification of DEGs and their corresponding metabolic pathways after sample treatment. In this study, the transcriptomics analysis identified the following major signaling pathways through GO and KEGG enrichment analysis of DEGs among the control, model, and CA-C groups: TLRs signaling pathway, MAPK signaling pathway, RIG-I-like receptor signaling pathway, C-type lectin receptor signaling pathway, etc. Combined with the above findings, we selected genes in the TLR signaling pathway and the downstream MAPK and PI3K/AKT signaling pathways to verify the action targets of CA-C using the RT-qPCR method.

TLRs work via two pathways: MyD88-dependent and MyD88-independent. In the MyD88-dependent pathway, TLR ligand binding recruits MyD88 to the Toll/Interleukin-1 receptor (TIR) domain, leading to the phosphorylation of interleukin-1 receptor-associated kinase (IRAKs) and activation of downstream molecules like tumor necrosis factor receptor-associated factor 6 (TRAF6). These signaling events culminate in the nuclear translocation of inflammatory transcription factors, including nuclear factor-κB (NF-κB) and activator protein-1 (AP-1), thereby promoting the transcription of pro-inflammatory genes [29]. TLRs are natural immune receptors that can participate in cells’ immune and inflammatory responses by recognizing ligands. There is also a close link between the inflammatory response and thrombosis. Studies have shown that activation of platelet TLRs has a strong effect on platelet activation and aggregation, which in turn promotes thrombosis. Activation of platelets triggers autoinflammation to release inflammatory mediators, as well as active molecules like thrombin, and platelets recognize danger signals from pathogens and cellular damage by expressing TLRs on their surface and inside [30]. The activation of platelet TLR4/nod-like receptor protein 3 (NLRP3) signaling plays a critical role in up-regulating platelet aggregation and interferes with perfusion recovery in muscle ischemia. [31]. In addition, platelet TLR4 was found to mediate cellular fibronectin containing extra domain A (Fn-EDA+) to promote thrombus formation [32]. Mice with platelet TLR4-specific deficiency had a prolonged time to first thrombosis and complete occlusion [32]. Besides, NF-κB is a key downstream mediator of TLR2 and TLR4-mediated platelet effects [33]. Both TLR4 and TLR2 receptors could regulate tissue factor (TF) expression through activation of AP-1 and NF-κB kinase pathways induced by thrombosis [34]. The role of TLR5 in atherothrombotic diseases has also been the focus of many studies [35]. Our research proved that the mRNA expressions of genes *tlr2*, *tlr4ab*, *tlr5*, *myd88*, *nf-κb*, *ap-1*, and *IL-1β* were markedly suppressed by CA-C in thrombotic zebrafish. This finding suggested that the mechanism of CA-C’s antithrombotic activity was related to the inhibition of the TLR signaling pathway and inflammatory responses.

Multiple downstream signaling pathways of platelet TLRs are directly or indirectly involved in thrombus development. In platelets, TLRs interact with MyD88 and TIRAP to stimulate downstream signaling pathways, including interactions between IRAK1/ IRAK4 and TRAF6 and activation of JNK and PI3K/AKT pathways. Other related signaling pathways, such as p38MAPK and ERK1/2, can also be activated [30]. Regulation of TLR4/MyD88 and cGMP/PKG pathways has been shown to be able to stimulate platelet secretion and enhance platelet activation [36]. Besides, the TLR4-PI3K/Akt-extracellular regulated protein kinases 1/2 (ERK1/2)-phospholipase A2 (PLA2) pathway, which is dependent on the formation of reactive oxygen species (ROS) and thrombospondane A2 (TXA2), can promote human platelet activation [37]. TLR2 can also activate platelets and inflammatory pathways to form thrombus through the PI3K/AKT signaling pathway. PI3K/AKT regulates TLR2-mediated platelet functional responses, including platelet aggregation, adhesion, secretion, leukocyte interactions, and ROS production [38]. Additionally, studies have shown that von Willebrand factor (vWF) can induce platelet aggregation, initiating platelet activation at the site of damaged vessels [39]. The PI3K*β*, a major mediator of the effects of α_IIb_β_3_ in platelet activation, stimulates Akt phosphorylation upon stimulation of the vWF receptor glycoprotein Ib-IX [40]. By RT-qPCR analysis, we found that CA-C treatment reduced the expression levels of genes *map2k2b* (MEK), *map2k7* (MKK), *mapk1* (ERK), *mapk14* (p38), *mapk10* (JNK), *akt2* (AKT), *pik3cb* (PI3K), and *vwf* in AA-induced thrombus zebrafish, suggesting the inhibitory effects of CA-C on MAPK and PI3K/AKT signaling pathways.

Based on the aforementioned results, CA-C can alleviate AA-induced thrombus in zebrafish by regulation of TLRs and their downstream pathways, MAPK and PI3K/AKT signaling pathways (Figure 10). Increasing evidence suggests that TLRs and their downstream pathways are involved in a variety of cardiovascular pathophysiologies, such as atherosclerosis and hypertension, indicating the value of targeting the TLR system in the treatment to cardiovascular diseases. More effects of CA-C on other cardiovascular diseases need to be further developed. Besides, this study still has several limitations: (1) the unidentified constituents in CA-C still require systematic chemical isolation and structure identification; (2) TLRs-related pathways deserve more systematic mechanism studies and target validation by western blotting assay, gene editing, and other methods; (3) The activities and potential molecular mechanisms of many potential bioactive components in CA-C need to be further evaluated.

## 4. Materials and Methods

### 4.1. Chemicals and Regents

Dimethyl sulfoxide (DMSO) was purchased from Shanghai Aladdin Biochemical Technology Co., Ltd. (Shanghai, China). Arachidonic acid and Macropore adsorptive resin D101 were purchased from Beijing Soleberg Technology Co., Ltd. (Beijing, China). Aspirin was purchased from Shanghai Yuanye Biotechnology Co., Ltd. (Shanghai, China). HPLC-grade methanol, analytical-grade ethanol, analytical-grade petroleum ether, and methylene blue were purchased from Sinopharm Chemical Reagent Co., Ltd. (Shanghai, China). FastPure Cell/Tissue Total RNA lsolation Kit-BOX 2 (RC101-01), HiScript^®^ Ⅲ RT SuperMix for qPCR (+gDNA wiper) (R323-01) and ChamQ Universal SYBR qPCR Master Mix (Q711-02) were provided by Vazyme Biotech Co., Ltd. (Nanjing, China).

### 4.2. Plant Material

*C. album* was collected in August 2023 in Lixia District, Jinan, Shandong Province, China, and identified by Jinchuan Zhou (College of Pharmacy, Linyi University). A voucher specimen (No. 20230817) has been deposited at the Key Laboratory for Drug Screening Technology of Shandong Academy of Sciences (Jinan, China).

### 4.3. Extraction and Separation

50 kg (wet weight) of *C. album* aerial parts were harvested and placed in a temperature drying oven at 35 °C for 48 h. After drying, the samples were ground into a fine powder and passed through a 40-mesh sieve, yielding 10 kg of powdered material. A 95% (*v*/*v*) ethanol solution was used with a material–liquid ratio of 1:5 (g/mL) to extract plants by maceration at room temperature for 45 days. Post filtration, the combined filtrates were concentrated under reduced pressure, resulting in the ethanol extract CA (741 g, 7.4% extraction rate). CA was suspended in water and defatted with the same volume of petroleum ether four times to obtain the water residue layer (519 g). It was then separated by chromatography on a column of D101 macroporous adsorbent resin, eluting with EtOH-H_2_O (0%, 30%, 60%, and 95% (*v*/*v*)) to acquire four fractions respectively, CA-A (242 g), CA-B (34 g), CA-C (42 g), and CA-D (55 g).

### 4.4. UPLC-Q-TOF-MS Analysis

UPLC-Q-TOF-MS analysis was performed on an Agilent 1290 UPLC and an Agilent 6545 LC/Q-TOF spectrometry system, with an Agilent ZORBAX RRHD SB-Aq column (2.1 × 100 mm, 1.8 µm) at 30 °C. The mobile phase included acetonitrile (A) and 0.1% formic acid aqueous solution (B). The following solvent gradient was adopted: 5–10% A (0–5 min), 10–15% A (5–10 min), 15% A (10–18 min), 15–30% A (18–25 min), 30–45% A (25–30 min), 45–55% A (30–40 min), 55–70% A (40–50 min), 70–90% A (50–55 min), 90% A (55–57 min), 90–5% A (57–57.1 min), 5% A (57.1–60 min), with the flow rate of 0.3 mL/min. The settings used for the MS analysis were as follows: ESI-Negative/Positive ion mode, mass spectrometry parameters were Mass Range, 50-1700, Gas Temp, 325 °C, Nebulizer, 45 psi, Vcap, 4000 V, Nozzle Voltage, 1000 V, Fragmentor, 175 V, Skimmer, 60 V. The mass spectrometry data were matched to the MassHunter PCDL Manager (version B.08.00) database to identify compounds and to identify compounds not included in the database based on literature reports, mass spectrometry cleavage mechanism, and so on.

### 4.5. Zebrafish Maintenance

The AB wild-type zebrafish was provided by the Engineering Research Center of Zebrafish Models for Human Diseases and Drug Screening of Shandong Province. The embryos were incubated with fish water at 28 °C, E3 medium (0.29 g/L NaCl, 0.013 g/L KCl, 0.048 g/L CaCl_2_, 0.082 g/L MgCl_2_, pH 7.2) was used to culture the zebrafish. Embryos were obtained through natural spawning. All zebrafish experiments in this study complied with the standard ethical guidelines and were ratified by the Experimental Animal Welfare Ethics Committee of the Biology Institute of Shandong Academy of Science. The ethics permit number is SWS20231202.

### 4.6. Drug Safety Testing

Zebrafish with normal development at 72 h post-fertilization (hpf) were selected and randomly transferred into a 24-well plate with ten fish per well. Three replicate wells were set up. Zebrafish in different groups were treated with different concentrations of CA at 400, 200, 100, 80, 60, 40, 20, and 0 μg/mL or CA-C at 600, 400, 200, 100, 50, 25, and 0 μg/mL for 24 h, respectively. The death of zebrafish was observed and photographed using an Olympus microscope (Olympus, Tokyo, Japan). The absence of a heartbeat was used as a criterion to determine the death of zebrafish. The lethality curve was plotted using concentrations of the sample as the horizontal coordinate and mortality as the vertical coordinate to calculate the 1% lethal concentration (LC_1_).

### 4.7. Antithrombotic Effect Assays

#### 4.7.1. Cardiac Erythrocyte Assay

The 72 hpf AB wild-type zebrafish larvae with normal development were selected and randomly grouped into 24-well plates with ten fish per well. Four kinds of groups were randomly set as follows: blank control group (zebrafish culture water), model group (AA), positive control group (AA + aspirin), and sample groups with different doses (AA + 12.5, 25 and 50 μg/mL of CA and CA-C for their activity evaluation, AA + 50 μg/mL of CA and CA-A–D for activity comparison). The positive control group and sample groups had 22.5 μg/mL of aspirin added, and the samples were tested 6 h in advance. For 1.5 h, all groups except the blank control group were treated with AA solution at a final concentration of 80 μM. Each group except the blank control group was treated for 1.5 h with 80 μM AA solution. A 15-min light-protected staining protocol was applied to each group of zebrafish using O-dianisidine dye liquor at 1.0 mg/mL. Zebrafish were washed three times with water, fixed with 4% paraformaldehyde, and photographed under an Olympus microscope. The zebrafish cardiac erythrocyte staining area (reflecting the number of cardiac erythrocytes) was measured and calculated using the Image-Pro Plus 5.0 software (Media Cybernetics, Inc., Bethesda, USA). All treatments were performed in triplicate.

#### 4.7.2. Caudal Thrombus Assay

The experimental scheme was the same as that in Section 4.7.1. After staining, rinsing, and fixation, Olympus microscopes were used to capture caudal images, measuring the thrombus area in the caudal of the zebrafish and calculating its size with Image-Pro Plus 5.0.

#### 4.7.3. Assay for Blood Flow Dynamics of the Caudal Vein

The experimental grouping and drug treatment were the same as those in Section 4.7.1. Zebrafish were placed in a lateral position onto methylcellulose-coated slides. The blood flow video was recorded for 10 s from the postcloacal vessels of zebrafish using the Zebralab blood flow system (ViewPoint, Lyon, France). Then, the video was analyzed for blood flow velocity using the MicroZebraLab BloodFlow 3.4.6 software (ViewPoint, Lyon, France), which generated the blood flow rate per frame by detecting the change in pixel density and combining it with the blood vessel flow rate diameter in nL/s. Blood flow velocities in zebrafish tails were statistically analyzed for each group, and hemodynamic pictures were obtained.

### 4.8. Network Pharmacology Analysis

Based on the UPLC-Q-TOF-MS compositional analysis results, the compounds in the CA-C fraction were screened out. The structural formulas of the compounds (sdf format) were obtained through the PubChem database and imported into Swiss ADME (swisstargetprediction.ch) (accessed on 14 June 2024) and PharmMapper (pharmmapper/index.html) (accessed on 14 June 2024) databases to gain component-related targets. Disease-related targets were searched from GeneCards (https://www.genecards.org) (accessed on 14 June 2024) and DrugBank (https://www.drugbank.ca) (accessed on 23 June 2024) with the keyword “thrombosis”. Component and disease-associated targets were imported into the Venn database (https://bioinformatics.psb.ugent.be/webtools/Venn/) (accessed on 26 June 2024) to obtain intersection targets. After obtaining the intersection targets, a protein-protein interaction (PPI) network was constructed using STRING 11.0 (https://string-db.org/) (accessed on 26 June 2024) with species parameters restricted to “Homo sapiens” and a confidence cut-off value set at 0.95. Gene Ontology (GO) and Kyoto Encyclopedia of Genes and Genomes (KEGG) enrichment analyses were performed on these intersection targets using the Metascape database (http://metascape.org/gp/index.html) (accessed on 28 June 2024) with a *p*-value threshold of at least 0.01. Based on the component-target relationship and the target-pathway relationship, the “component-target-pathway” network was visualized by Cytoscape 3.9.1 software.

### 4.9. mRNA-Sequencing and Bioinformatics Analysis

The 72 hpf AB wild-type zebrafish larvae with normal development were selected and randomly grouped into 6-well plates with 30 fish per well. According to the methods of experimental grouping and drug treatment described in Section 4.7.1, zebrafish of the control group, the model group, and the sample group treated with 50 μg/mL of CA-C were collected, respectively, and stored at −80 °C. Three replicates of each treatment were subjected to RNA sequencing. A TRIzol Kit (Invitrogen, Carlsbad, CA, USA) was used for total RNA extraction following the manufacturer’s protocol. A Bioanalyzer (Agilent Technologies, Santa Clara, CA, USA) was used for RNA integrity analysis. Transcriptome libraries were prepared with the VAHTS Universal V5 RNA-seq Library Prep kit and sequenced on the Illumina Novaseq 6000 platform (Illumina Technologies, San Diego, CA, USA), producing 150 bp paired-end sequences. Differentially expressed genes (DEGs), which are defined by the thresholds of *p*-value < 0.05 and foldchange > 2 or foldchange < 0.5, were analyzed using DESeq2 1.20.0 software. GO enrichment (https://ftp.ncbi.nih.gov/gene/DATA/gene2go.gz) (accessed on 16 August 2024) and KEGG pathway analysis (https://www.genome.jp/kegg/) (accessed on 16 August 2024) of DEGs were performed to identify the significantly enriched GO terms and pathways. We used the R programming language (https://www.r-project.org/) (accessed on 16 August 2024) on the OECloud platform (https://cloud.oebiotech.com/task/) (accessed on 16 August 2024) to draw the volcano map and cluster heatmap.

### 4.10. RNA-Isolation and RT-qPCR

The 72 hpf AB wild-type zebrafish larvae with normal development were selected and randomly grouped into 6-well plates with 30 fish per well; zebrafish samples in each group were prepared and collected. Following the bioinformatics analysis, pivotal DEGs were selected for RT-qPCR validation. The bioinformatics analysis and pivotal DEGs were selected for RT-qPCR validation. Total RNA was isolated from the fresh fish samples employing the FastPure Cell/Tissue Total RNA Isolation Kit-BOX 2. Subsequently, cDNA synthesis was performed using the HiScript^®^ III RT SuperMix (Vazyme, Nanjing, China) for quantitative PCR, which includes a genomic DNA removal step. The expression levels of genes *tlr2*, *tlr4ab*, *tlr5*, *myd88*, *mapk1*, *mapk14*, *mapk10*, *map2k2b*, *map2k7*, *akt2*, *pik3cb*, *nf-kb*, *ap-1*, *IL-1β*, and *vwf* were identified by ChamQ Universal SYBR qPCR Master Mix, respectively. LightCyler 96 system (Roche, Basel, Switzerland) was used for RT-qPCR experiments; the reaction conditions were as follows: 30 s at 95 °C, followed by 40 cycles of 95 °C for 15 s and 60 °C for 30 s. *Rpl13a* was used as an internal control. RT-qPCR of each gene was performed in four duplications using the BIO-RAD CFX96 Real-Time System (Roche, Basel, Switzerland). Using the 2^−∆∆Ct^ equation, the relative quantity of transcripts was estimated. Table 2 reveals the sequences of all primers.

### 4.11. Statistical Analysis

In the experiments, data are presented as the mean ± standard deviation (SD). The comparison between the control group and multiple dose groups was performed using one-way ANOVA, post hoc analyses using the Tukey (HSD) test, and homogeneity of variance using the Bartlett test. All statistical analyses were conducted using GraphPad Prism 9 software (Version 8.1.1, San Diego, CA, USA). Results with *p*-values less than 0.05 were considered statistically significant.

## 5. Conclusions

In our study, we explored the new therapeutic effects of *C. album* on thrombosis. We determined the active fraction CA-C mainly responsible for the antithrombotic activity of this herb by AA-induced zebrafish model. Fifty-eight constituents of CA-C were detected using UPLC-Q-TOF/MS, of which most compounds were identified as alkaloids, steroidal saponins, and flavonoid components. Network pharmacology results showed that (2S,3R,10R,13R,14S)-2,3,14-trihydroxy-10,13-dimethyl-17-[(2R,3R,5R)-2,3,5-trihydroxy-5,6-dimethylheptan-2-yl]-2,3,4,5,9,11,12,15,16,17-decahydro-1H-cyclopenta[a]phenanthren-6-one, 9,12,13-trihydroxy-10,15-octadecadienoic acid, N-acetyl-DL-tryptophan, forskoditerpenoside C, kaempferol-3-O-rutinoside, azukisaponin III, β-ecdysone, turkesterone, rutin, and xanthorhamnin, etc. may be the main active components of CA-C for the treatment of thrombosis in zebrafish. Move forward mechanism research of CA-C, through a comprehensive strategy combining network pharmacology and transcriptomics analysis, together with experimental verification of RT-qPCR, revealed that CA-C exerted a significant antithrombotic effect by mediating the TLR signaling pathway and its downstream MAPK and PI3K/AKT signaling pathways. This study broadens the clinical application of plant *C. album* and provides a new insight into the chemical profile, pharmacodynamics and potential mechanisms of CA-C as candidate agents for the treatment of thrombosis.

## Figures and Tables

**Table 1:** Chemical constituents identified from CA-C using UPLC-Q-TOF-MS.

NO.	RT (min)	Compound	Areas of Peak	Percentage of Peak Area	Error (ppm)	Molecular Formula	Molecular Weight	Classification
1	7.33	Unidentified	418,613	0.18%	−1.2	C_24_H_14_N_2_O_4_	394.1	Alkaloid
2	9.68	Unidentified	5,075,087	2.14%	4	C_20_H_28_N_2_O_4_	360.2	Alkaloid
3	10.23	N-Acetyl-DL-tryptophan	12,821	0.01%	−0.2	C_13_H_14_N_2_O_3_	246.1	Amino acid derivatives
4	11.56	Unidentified	575,710	0.24%	7.5	C_17_H_16_N_4_O_2_	308.13	Alkaloid
5	11.84	Ochangoside analogues	22,853	0.01%	5	C_20_H_14_N_4_O_6_	406.09	Alkaloid
6	12.52	Sasanquin or isomer	2,293,896	0.97%	0	C_21_H_30_O_11_	458.18	Sugar derivatives
7	13.01	Unidentified	1,382,987	0.58%	−0.5	C_15_H_20_O_5_	280.13	/
8	13.59	Camellianoside or isomer	411,681	0.17%	2.1	C_32_H_38_O_20_	742.2	Flavonoid
9	14.36	Turkesterone	3,016,210	1.27%	2.3	C_27_H_44_O_8_	496.3	Steroid
10	14.78	β-Ecdysone	22,736,901	9.55%	3	C_27_H_44_O_7_	480.31	Steroid
11	14.97	Manghaslin or isomer	3,858,890	1.62%	1.7	C_33_H_40_O_20_	756.21	Flavonoid
12	15.6	Rutin	11,197,633	4.70%	0.4	C_27_H_30_O_16_	610.15	Flavonoid
13	16.24	Unidentified	4,202,950	1.77%	0.4	C_19_H_32_O_7_	372.21	/
14	16.8	Ochangoside analogues	3,428,915	1.44%	4.4	C_22_H_16_N_4_O_7_	448.1	Alkaloid
15	17.06	β-Ecdysone isomer	2,751,005	1.16%	−1.1	C_27_H_44_O_7_	480.31	Steroid
16	17.87	Unidentified	2,539,013	1.07%	−0.3	C_33_H_54_O_11_	626.37	Saponin
17	18.99	Quercetin 3-xylosyl-(1->2)-alpha-L-arabinofuranoside	8,081,510	3.39%	2	C_25_H_26_O_15_	566.13	Flavonoid
18	20.17	(2S,3R,10R,13R,14S)-2,3,14-Trihydroxy-10,13-dimethyl-17-[(2R,3R,5R)-2,3,5-trihydroxy-5,6-dimethylheptan-2-yl]-2,3,4,5,9,11,12,15,16,17-decahydro-1H-cyclopenta[a]phenanthren-6-one or isomer	7,280,938	3.06%	1.6	C_28_H_46_O_7_	494.32	Steroid
19	20.44	(2S,3R,10R,13R,14S)-2,3,14-Trihydroxy-10,13-dimethyl-17-[(2R,3R,5R)-2,3,5-trihydroxy-5,6-dimethylheptan-2-yl]-2,3,4,5,9,11,12,15,16,17-decahydro-1H-cyclopenta[a]phenanthren-6-one or isomer	7,143,672	3.00%	1.4	C_28_H_46_O_7_	494.32	Steroid
20	20.46	Kaempferol-3-O-rutinoside or isomer	2,585,993	1.09%	−0.8	C_27_H_30_O_15_	594.16	Flavonoid
21	21.21	Xanthorhamnin or isomer	548,948	0.23%	−1.5	C_34_H_42_O_20_	770.23	Flavonoid
22	21.73	Ochangoside analogues	616,553	0.26%	3.8	C_23_H_26_N_4_O_6_	454.19	Alkaloid
23	22.02	Unidentified	1,164,620	0.49%	−0.3	C_18_H_34_O_9_	394.22	/
24	22.32	Ochangoside analogues	77,581	0.03%	3.2	C_23_H_24_N_4_O_6_	452.17	Alkaloid
25	23.21	Ajugasalicioside F or isomer	1,794,673	0.75%	0.9	C_35_H_58_O_12_	670.39	Saponin
26	23.34	Unidentified	3,569,670	1.50%	3.6	C_34_H_44_O_15_	692.27	Steroid
27	23.63	Unidentified	3,252,710	1.37%	1.8	C_24_H_42_O_14_	554.26	/
28	24.03	Unidentified	1,485,841	0.62%	0.1	C_26_H_52_O_10_	524.36	/
29	24.49	Unidentified	2,092,369	0.88%	0.7	C_33_H_54_O_10_	610.37	/
30	24.86	Unidentified	1,877,497	0.79%	1.3	C_41_H_70_O_17_	834.46	Saponin
31	25.15	Unidentified	1,662,002	0.70%	1.6	C_41_H_68_O_17_	832.45	Saponin
32	25.3	Unidentified	2,308,288	0.97%	1.3	C_24_H_41_NO_9_	487.28	Alkaloid
33	25.52	Azukisaponin III or isomer	281,837	0.12%	−0.4	C_42_H_66_O_15_	810.44	Saponin
34	25.65	Ochangoside analogues	8,168,834	3.43%	6.5	C_22_H_26_N_4_O_7_	458.18	Alkaloid
35	25.93	Unidentified	16,793,342	7.05%	6.6	C_22_H_26_N_4_O_7_	458.18	Alkaloid
36	26.29	Unidentified	6,432,661	2.70%	2.8	C_19_H_21_NO_5_	343.14	Alkaloid
37	26.62	Ochangoside analogues	7,985,342	3.35%	6.7	C_22_H_26_N_4_O_7_	458.18	Alkaloid
38	27.09	Ochangoside analogues	11,309,288	4.75%	7.4	C_22_H_26_N_4_O_7_	458.18	Alkaloid
39	27.32	9,12,13-Trihydroxy-10,15-octadecadienoic acid or isomer	2,227,110	0.94%	1.9	C_18_H_32_O_5_	328.22	Fatty acid
40	27.49	9,12,13-Trihydroxy-10,15-octadecadienoic acid or isomer	2,072,285	0.87%	2	C_18_H_32_O_5_	328.22	Fatty acid
41	27.89	9,12,13-Trihydroxy-10,15-octadecadienoic acid or isomer	3,938,724	1.65%	2.5	C_18_H_32_O_5_	328.22	Fatty acid
42	28.03	9,12,13-Trihydroxy-10-octadecenoic acid or isomer	12,151,177	5.10%	2.8	C_18_H_34_O_5_	330.24	Fatty acid
43	28.35	9,12,13-Trihydroxy-10,15-octadecadienoic acid or isomer	2,951,132	1.24%	2.2	C_18_H_32_O_5_	328.22	Fatty acid
44	28.6	9,12,13-Trihydroxy-10,15-octadecadienoic acid or isomer	1,480,947	0.62%	0.8	C_18_H_32_O_5_	328.22	Fatty acid
45	28.83	Unidentified	2,243,838	0.94%	1.3	C_34_H_44_O_11_	628.29	/
46	29.16	Spinasaponin A or isomer	347,8076	1.46%	1.2	C_42_H_66_O_14_	794.45	Saponin
47	29.41	Unidentified	955,201	0.40%	4.6	C_30_H_42_O_12_	594.27	/
48	29.53	Unidentified	710,610	0.30%	5.2	C_30_H_42_O_12_	594.27	/
49	29.82	(3β,12α)-12-Hydroxy-28-oxo-13,28-epoxyoleanan-3-yl α-D-mannopyranosiduronic acid or isomer	878,366	0.37%	−0.6	C_36_H_56_O_10_	648.39	Saponin
50	30.32	Unidentified	772,582	0.32%	−2.7	C_23_H_44_O_19_	624.25	Saponin
51	30.9	Unidentified	437,050	0.18%	−2.3	C_17_H_28_O_5_	312.19	/
52	31.53	Trp-lys-asp-phe or isomer	1,046,036	0.44%	−1.3	C_30_H_38_N_6_O_7_	594.28	Peptide
53	31.86	Asp-trp-phe-lys or isomer	1,424,328	0.60%	−1.5	C_30_H_38_N_6_O_7_	594.28	Peptide
54	32.53	Unidentified	13,602,821	5.71%	6.4	C_30_H_42_O_11_	578.27	/
55	33.67	Unidentified	2,948,779	1.24%	5.2	C_30_H_44_O_11_	580.29	Sugar derivatives
56	34.19	Forskoditerpenoside C or isomer	20,829,413	8.75%	6.5	C_28_H_44_O_11_	556.29	Diterpenoid
57	35.45	Unidentified	1,711,740	0.72%	8.1	C_20_H_24_O_3_	312.17	Cyclopentanoperhydro-phenanthrene
58	37.54	Unidentified	1,404,050	0.59%	6.2	C_21_H_26_O_3_	326.19	Cyclopentanoperhydro-phenanthrene

**Table 2:** Primer sequences for each gene.

Primer Name	Forward Primer (5′→3′)	Reverse Primer (3′→5′)
*Rpl13a*	AGAGCTATGAGCTGCCTGACG	CCGCAAGATTCCATACCCA
*tlr2*	TGCTGTCGGTCGATTACCTG	ACACAGGGAAAACGAAGGCT
*tlr5*	GCAACAAACACCAGGACTCG	CGCGCCCGTCTCTAATTGTA
*tlr4ba*	TGCTGCGAGAGATCGGAAGAAA	ACAAAAGCAGATCGAATATCCAGC
*map2k7*	CATCGTGATCCAGCTCAGTCC	TGAGGGGAGCTTTCTGACGA
*ap-1*	CCACCGCTCTCTCCTATC	ATCCTCTCCAGTTTCCTCTT
*mapk10*	CATTCTGGGCATGGGCTACA	CCTGCCGGGGAATAGGATTTT
*vwf*	GTTCCTGCTGAGAGCGTCTT	ACGACCGATGTTTGTGCTCT
*myd88*	ACGGCTAATCCCTGTCGTCT	CAGATGGTCAGAAAGCGCAG
*mapk1*	TACGTCTGGTGCTCAAACGG	AATGCGTCCCGACAGAGACA
*mapk14*	AACGTGACGGTGGACATTTG	AGCTGATGTAAGTACGAGCCT
*pik3cb*	GGAGGCGCAGACATATCCTC	AGAACAGGCAGGAATGGTCG
*akt2*	TAAGCAGACGTGAGCAGGTG	AAGCTCGTTCCACCCTTCAA C
*map2k2b*	CTCTAAAAGGCCAAACGCCC	AGTTGTGTGTGGTGGTCTCC
*nf-κb*	ATAGATGAGAACGGAGACACG	CGCTACTAACTTGGGTTGCTT
*IL-1β*	GTGGACTTCGCAGCACAAAA	AAGACGGCACTGAATCCACC

## Data Availability

The data presented in this study are available on request from the corresponding author.

## Abbreviations

The following abbreviations are used in this manuscript:

UPLC-Q-TOF-MS	ultra-high performance liquid chromatography-quadrupole time-of-flight tandem mass spectrometry
TLRs	toll-like receptors
MAPK	mitogen-activated protein kinase
KEGG	Kyoto Encyclopedia of Genes and Genomes
PPI	protein–protein interaction
LC_1_	1% lethal concentration
AA	arachidonic acid
GO	Gene Ontology
TCM	traditional Chinese medicine
DEGs	differentially expressed genes
PI3K	phosphoinositide-3-kinase
RT-qPCR	real-time quantitative PCR
PKC	protein kinase C
TXB2	thromboxane B2
vWF	von Willebrand factor
NF-κb	nuclear factor-κB
ERK	extracellular regulated protein kinases
PLA2	phospholipase A2
JNK	c-Jun N-terminal kinase
TIR	toll/interleukin-1 receptor
AP-1	activator protein-1
MyD88	myeloid differentiation primary response protein 88
